# Layer-by-Layer Heparinization of the Cell Surface by Using Heparin-Binding Peptide Functionalized Human Serum Albumin

**DOI:** 10.3390/ma11050849

**Published:** 2018-05-20

**Authors:** Guowei Song, Yaning Hu, Yusheng Liu, Rui Jiang

**Affiliations:** College of Life and Health Sciences, Northeastern University, Shenyang, 110169, China; panda.song749@gmail.com (G.S.); hhhhhsherlock@gmail.com (Y.H.); liuyusheng5691@163.com (Y.L.)

**Keywords:** cell surface modification, heparin-binding peptide, heparinization, layer-by-layer, human serum albumin

## Abstract

Layer-by-layer heparinization of therapeutic cells prior to transplantation is an effective way to inhibit the instant blood-mediated inflammatory reactions (IBMIRs), which are the major cause of early cell graft loss during post-transplantation. Here, a conjugate of heparin-binding peptide (HBP) and human serum albumin (HSA), HBP-HSA, was synthesized by using heterobifunctional crosslinker. After the first heparin layer was coated on human umbilical vein endothelial cells (HUVECs) by means of the HBP-polyethylene glycol-phospholipid conjugate, HBP-HSA and heparin were then applied to the cell surface sequentially to form multiple layers. The immobilization and retention of heparin were analyzed by confocal microscopy and flow cytometry, respectively, and the cytotoxity of HBP-HSA was further evaluated by cell viability assay. Results indicated that heparin was successfully introduced to the cell surface in a layer-by-layer way and retained for at least 24 h, while the cytotoxity of HBP-HSA was negligible at the working concentration. Accordingly, this conjugate provides a promising method for co-immobilization of heparin and HSA to the cell surface under physiological conditions with improved biocompatibility.

## 1. Introduction

Transplantation of therapeutic cells such as pancreatic islets or mesenchymal stem cells is a promising therapy for a variety of difficult diseases, and it shows several advantages over whole-organ transplantation. One of the major challenges of this treatment is that once these therapeutic cells contact the recipients’ blood, an innate immune response called instant blood-mediated inflammatory reaction (IBMIR) will be triggered by both coagulation and complement systems of recipient, which is followed by a rapid binding of platelets and infiltration of leukocytes into the clot, resulting in great loss of transplanted cells and a significant influence on the clinical results of transplantation. Thus, to minimize the loss of graft in the post-transplantation period, various protective strategies to inhibit IBMIR have been developed in the past several years.

Previous studies showed that the systemic administration of several anticoagulant agents such as melagatran [1], activated protein C [2] and low molecular weight dextran sulfate [3], could reduce or inhibit IBMIR. However, the systemic administration of anticoagulants leads an increased risk of bleeding, especially in patients with impaired liver and kidney functions. To resolve this problem, many attempts have been made to avoid systemic treatment, and researchers found that IBMIR was effectively inhibited by coating islet cells with a variety of anti-thrombotic as well as anti-inflammatory agents, such as recombinant thrombomodulin [4], urokinase [5,6], soluble domain of complement receptor 1 (sCR1) [7], and heparin [8]. In our previous work, a major endothelial anticoagulant protein, thrombomodulin (TM), was immobilized site-specifically onto different surfaces via bioorthogonal reactions such as “click chemistry” or Staudinger ligation [9,10,11,12]. Immobilized TM forms an endothelium-mimicking layer with anticoagulant activities; however, the coating processes of these methods are complicated for the treatment of living cells, and these methods only afford a single protein layer on the cell surface. Thus, to explore a simplified method with improved capacities of anticoagulant agents is significant for cell surface modifications.

**Scheme 1:**
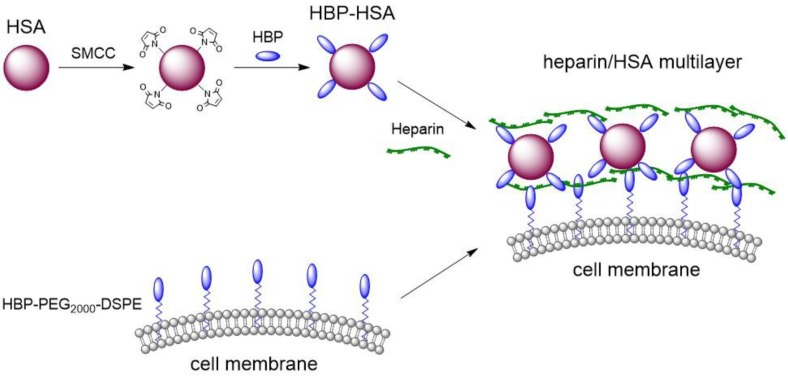
Illustration of layer-by-layer heparinization of the cell surface by using the HBP-HSA conjugate. HSA was randomly modified with multiple HBP molecules by using an SMCC crosslinker.

Heparin is a naturally occurring glycosaminoglycan, with a molecular weight ranging from 3 to 30 kDa. Unfractionated heparin (UFH) is the most extensively used anticoagulant in clinical practice, and its anti-thrombotic/anti-inflammatory activities make it an excellent material for protection of transplanted cells. However, in the bloodstream, a limited amount of immobilized heparin could be easily neutralized by platelet factor 4 (PF4), which is released from activated platelets during IBMIR triggered platelet aggregation [13]. Based on the reasons mentioned above, layer-by-layer techniques have been studied to increase the amount and stability of immobilized heparin, and thereby improve the transplantation efficiency of cell grafts [5,14]. Recently, Asif et al. reported a cell surface heparinization strategy mediated by heparin-binding peptide (HBP) [15]. In their study, a dodecapeptide (NSAHRTRGRQRS) with low cytotoxicity was identified from several HBP candidates and further conjugated with polyethylene glycol (PEG)–phospholipid. The HBP-PEG-phospholipid conjugate could be incorporated into the lipid bilayer of the cell membrane through its hydrophobic lipid tail, and surface heparinization could be achieved by heparin-HBP interactions. Based on their study, the HBP mentioned above was further applied to layer-by-layer heparinization by conjugation with human serum albumin (HSA) in this paper, as illustrated in Scheme 1. HSA is the most abundant plasma protein with an approximate molecular weight of 67 kDa. The relative large protein size and abundant surface primary amines allow the HSA molecule to readily be functionalized with multiple short peptides simultaneously, and therefore makes HBP-HSA an ideal adhesive of heparin. After establishing the conjugation process, the efficiency for preparation of heparin multilayer, and the cytotoxicity of HBP-HSA were also be evaluated.

## 2. Materials and Methods

### 2.1. Materials

All solvents and reagents were purchased from commercial sources. Recombinant HSA, fluorescein isothiocyanate (FITC) and unfractionated heparin were obtained from Sigma-Aldrich (Saint Louis, MO, USA). Human antithrombin III was obtained from Haematologic Technologies Inc. (Essex Junction, VT, USA). Maleimide-PEG_2000_-DSPE was obtained from Ponsure Biotech (Shanghai, China). Succinimidyl 4-(*N*-maleimidomethyl)cyclohexane-1-carboxylate (SMCC) was obtained from Thermo Fisher Scientific (Waltham, MA, USA). Heparin binding peptide with an N-terminal cysteine (CNSAHRTRGRQRS) was synthesized by NJpeptide (Nanjing, China).

### 2.2. Cell Culture

Human umbilical vein endothelial cells (HUVECs) and complete endothelial cell medium (ECM) were purchased from Sciencell (Carlsbad, CA, USA) and maintained according to the manufacturers’ instructions. In brief, HUVECs (catalog #8000) maintained in ECM (catalog #1001) were plated onto a collagen-coated T25 flask in a 37 °C humidified atmosphere of 95% air and 5% CO_2_. The exponentially growing sub-confluent cells were used for all experiments at passages 6 to 8.

### 2.3. Synthesis of HBP-PEG-DSPE Conjugate

HBP-PEG-DSPE was prepared as previously reported [15]. Briefly, maleimide-PEG_2000_-DSPE (5 mg, 1.7 μmol) was mixed with HBP (5 mg, 3.67 μmol) in 500 μL of phosphate-buffer (pH 7.2), and the solution was further incubated at RT for 6 h to yield the HBP-PEG_2000_-DSPE conjugate.

### 2.4. Synthesis and Characterization of HBP-HSA Conjugate

SMCC solution (50 µL; 0.55 μmol, 3.7 mg/mL in DMSO) was mixed with 500 µL of HSA (0.075 μmol, 10 mg/mL) in phosphate-buffer (pH 7.2) and allowed to react at RT for 1 h. The unreacted SMCC was then removed by a a HiTrap desalting column (5 mL, GE) equilibrated with phosphate buffer (pH 7.0). Next, maleimide group-modified HSA was further incubated with HBP (3 mg, 2.2 μmol) at RT for 2 h. The reaction product was then purified by a HiTrap desalting column equilibrated with phosphate-buffered saline (100 mM sodium phosphate, 150 mM sodium chloride, pH 7.4). The purified HBP-HSA was analyzed by 10% SDS-PAGE, and the particle size of HBP-HSA was measured by using a Zetasizer Nano-S90 (Malvern Instruments Inc., Westborough, MA, USA) based on dynamic light scattering (DLS) at a fixed angle of 90°. Each sample was analyzed in triplicate.

### 2.5. Immobilization of HBP-HSA and Heparin on the Cell Surface

HUVECs were plated onto a gelatin-coated 6-well culture plate (1 × 10^6^ cells per well) at 37 °C for overnight prior to use. Firstly, cells were rinsed twice with PBS and were then incubated in a solution of HBP-PEG_2000_-DSPE (0.5 mg/mL) at RT for 30 min. After washing with PBS, the cells were incubated with UFH (5 mg/mL in PBS) for 10 min to introduce the first heparin layer. Subsequent layers were introduced by incubating heparinized cells with HBP-HSA and UFH alternately for 30 min at RT until the desired number of layers was achieved.

### 2.6. FITC Labeling of Human Antithrombin (AT)

AT was diluted to a concentration of 1 mg/mL in 150 μL of carbonate−bicarbonate buffer (pH 9.0), and 50 μL of freshly prepared FITC solution was added (2 mg/mL in DMSO). The reaction mixture was gently vortexed at RT for 2 h in the dark. After the coupling reaction, free FITC was removed by dialysis against PBS, and the FITC-AT conjugate was concentrated by using a centrifuge filter device (Millipore Inc., Burlington, MA, USA) with a molecular weight cutoff of 3000 Da.

### 2.7. Observation of FITC-AT Labeled Cells by Using Confocal Microscopy

The heparinization level of cells was visualized by confocal microscopy. Firstly, HUVECs were seeded on round cover slides (14 mm diameter) in a 24-well cell culture plate (1 × 10^5^ cells/mL) and allowed to adhere overnight. After the heparinization procedure described above, the cover slides were subsequently incubated with FITC-AT (50 μg/mL in PBS) and DAPI (10 μg/mL) at RT for 30 min. After rinsing with PBS twice, the cover slide sections were imaged using a confocal laser scanning microscope (TCS SP8, Leica Microsystems Inc., Buffalo Grove, IL, USA).

### 2.8. Flow Cytometry for Detection of Immobilized Heparin

Heparinized HUVECs (5 × 10^5^ cells) were detached by treatment of trypsin-EDTA and were collected by centrifugation (200 g, 5 min). The cells were then washed twice and resuspended in 100 μL of PBS. Next, 10 μL of FITC-AT (100 μg/mL in PBS) was added to cell suspensions, followed by a 40 min incubation at RT. Finally, the cells were centrifuged (200 g, 5 min) to remove unbound FITC-AT and resuspended in 500 μL of PBS. FITC-AT labeled cells were analyzed by a flow cytometer (Accuri C6, BD Biosciences Inc., San Jose, CA, USA) according to the manufacturer’s instructions, and the results were further analyzed by FlowJo software, version 10 (FlowJo LLC, Ashland, OR, USA).

### 2.9. Cell Viability Assay

The cytotoxicity of HBP-HSA was accessed using a Cell Counting Kit-8 assay (CCK-8, Beyotime Biotechnology Inc., Shanghai, China). Firstly, HUVECs were seeded in a 96-well plate at a density of 5 × 10^3^ cells/well in 100 μL of culture medium. After culturing for 12 h, the cells were treated with various concentrations of HSA-HBP for 1 h and 24 h, respectively. The cells were then cultured at 37 °C for 12 h, followed by the addition of 10 μL CCK-8 solution. After 1 h incubation at 37 °C, absorbance at 450 nm was measured by a microplate reader. All experiments were performed three times, and the results are presented as the mean ± standard deviation.

## 3. Results

### 3.1. Preparation and Characterization of the HBP-HSA Conjugate

**Figure 1:**
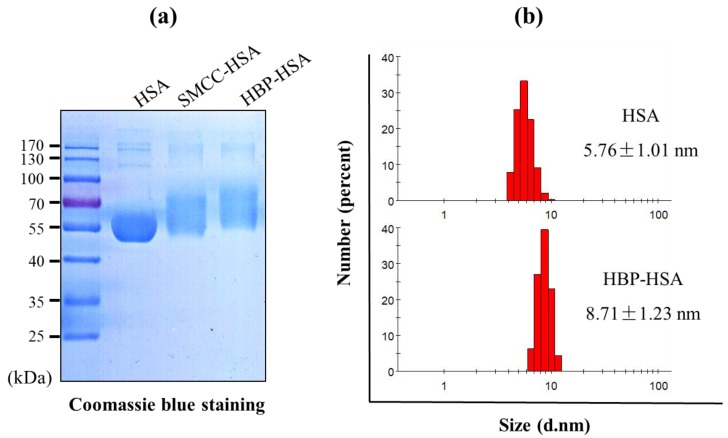
Synthesis and characterization of the HBP-HSA conjugate. (**a**) 12% SDS-PAGE analysis of the HBP-HSA conjugate with Coomassie blue staining. Lane 1, molecular weight marker; lane 2, SMCC modified HSA purified by a desalting column; lane 3, HBP conjugated with SMCC-HSA; (**b**) particle size histograms of HSA and HBP-HSA. Data for particle size represent the mean ± standard deviation of three measurements.

Firstly, HBP was conjugated with HSA by using the amine-to-sulfhydryl crosslinker SMCC. In this reaction, the primary amines of HSA were modified with SMCC at pH 7.2 for 1 h since the maleimide group of SMCC is unstable when the pH level is above 7.5. The reaction mixture was then applied on a Hitrap desalting column (GE) to remove excess SMCC, and the purified SMCC-HSA was subsequently reacted with the sulfhydryl group of the cysteine-containing HBP at pH 7.2; the unreacted HBP was also removed by the Hitrap desalting column, and the product HBP-HSA was obtained. The HSA and HSA conjugates were further analyzed by SDS-PAGE as shown in Figure 1a. The molecular weight of the recombinant HSA protein increased after SMCC modification (lane 2), and the conjugation of HBP to SMCC-HSA further increased the molecular weight of the latter (lane 3). Moreover, the broader protein bands of HSA conjugates indicated that multiple amines on HSA were randomly modified. The subsequent dynamic light scattering (DLS) analysis showed the particle size of HBP-HSA was significantly greater than that of HSA, which was consistent with the result of SDS-PAGE analysis (Figure 1b). Furthermore, the size distribution of HBP-HSA was homogeneous, and no aggregations were observed. After the purification process, the protein concentration of HBP-HSA was determined by BCA assay for further applications.

### 3.2. Immobilization of UFH and HBP-HSA on Cell Surface

**Figure 2:**
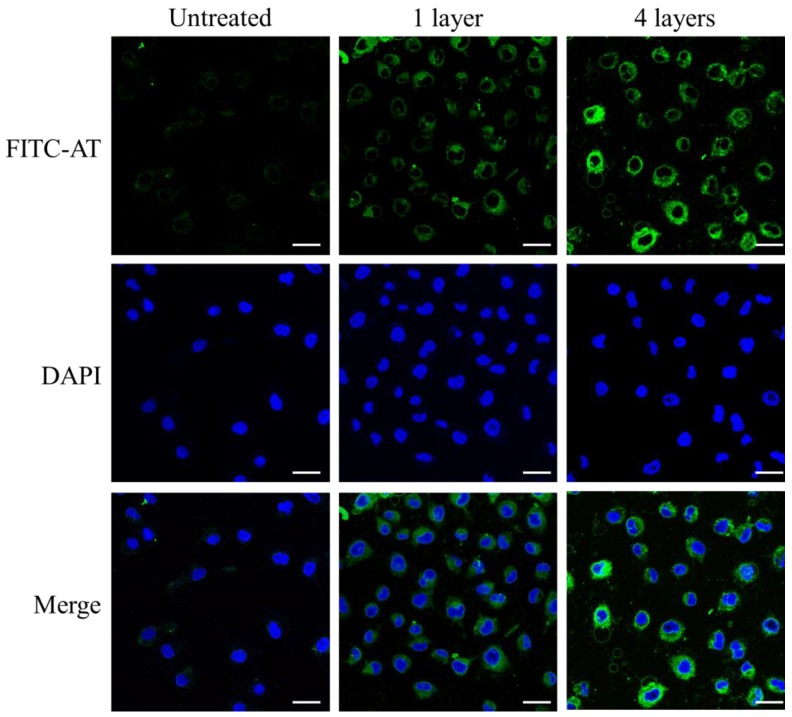
Confocal images of heparinized HUVECs. Cells were labeled with DAPI, and bound UFH was visualized by FITC–labeled antithrombin (FITC-AT). Scale bars represent 20 µm.

**Figure 3:**
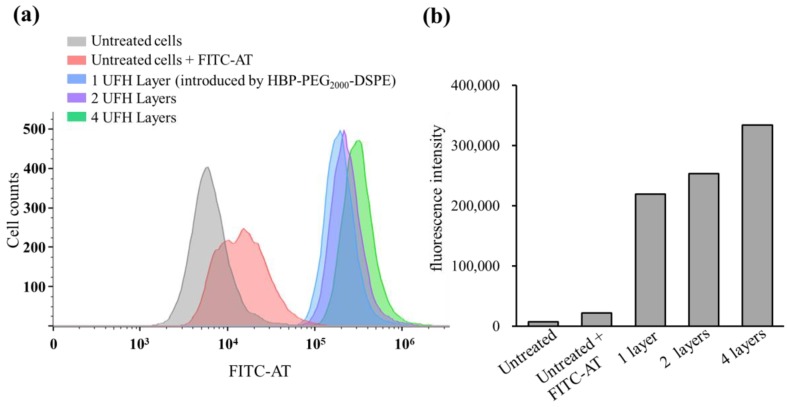
Flow cytometry analysis of heparin-coated HUVECs. Cells were coated with 1, 2 and 4 layers of UFH and then incubated with FITC-AT for 30 min. (**a**) FAC analysis of FITC-AT labeled cells; (**b**) histogram of mean fluorescence intensity of each sample.

Next, we selected human umbilical vein endothelial cells (HUVECs) as a model cell, and up to 4 layers of unfractionated heparin (UFH) were immobilized to test the feasibility and effectivity of this layer-by-layer strategy. The first layer of immobilized UFH on the cell surface was prepared by incubating HUVECs with the synthesized HBP-PEG_2000_-DSPE conjugate as described in the methods section. HBP-HSA and UFH were then alternately applied to HUVECs to form multilayers. The immobilized UFH could be detected via interactions between UFH and antithrombin (AT), and therefore FITC labeled AT (FITC-AT) was prepared for subsequent analysis. The successful heparinization was then visualized by confocal microscopy. As shown in Figure 2, the fluorescence of FITC-AT was clearly observed on the surface of HBP-conjugate treated cells, and fluorescence intensity was enhanced with the increase of heparin layers, whereas weak fluorescence was observed in control cells likely because the endothelial cell is one of the few cells that have weak heparin affinity. The complete endothelial cell culture medium contains trace amount of heparin to promote its growth, and thus the cell-binding heparin was detected as the background. Next, the flow cytometric method was utilized to accurately determine the coating efficiency. HUVECs with different numbers of heparin layers were incubated with FITC-AT for 30 min and then analyzed by a flow cytometer. A dramatic increase in immobilized UFH on the cell surface was revealed (Figure 3a), and it was found that the amount of UFH was positively correlated with an increase in the number of layers. The mean fluorescence intensity of each sample was plotted (Figure 3b), and the non-specific binding between HUVEC and UFH (column 2) was negligible compared with HBP-mediated heparinization, suggesting that the immobilization of UFH was specific and efficient.

### 3.3. Stability of Immobilized Heparin on the Cell Surface

**Figure 4:**
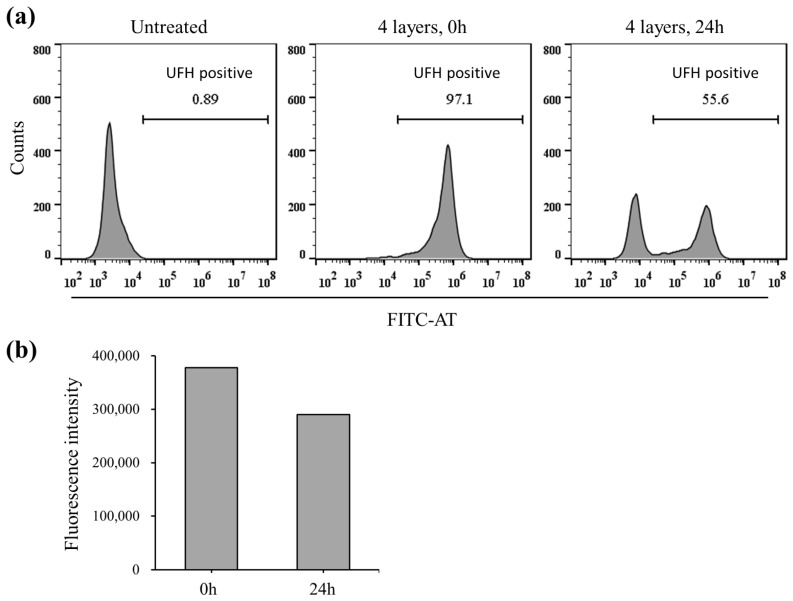
Flow cytometric analysis of UFH retention on cell surfaces. (**a**) HUVECs coated with 4 layers of UFH were cultured at 37 °C for 24 h, followed by incubation with FITC-AT for 30 min. The percentages of UFH-positive cells were labeled; (**b**) histogram of mean fluorescence intensities of UFH-positive cells.

In order to evaluate the retention of immobilized heparin on the surface of HUVECs, cells were heparinized with four layers of UFH as described above. After culturing in ECM for an additional 24 h at 37 °C, the cells were labeled with FITC-AT and then analyzed by a flow cytometer. As shown in Figure 4a, over 97 % of cells became UFH positive after heparinization treatment (middle panel), and after culturing for 24 h, the percentage of UFH-positive cells decreased to 56, which could be due to cell division during this period. In Figure 4b, the mean fluorescence intensities of UFH-positive cells at 0 h and 24 h were compared, and the histogram indicated that more than 80 % of UFH was retained on the cell surface after culturing for 24 h.

### 3.4. Cell Viability Assay

**Figure 5:**
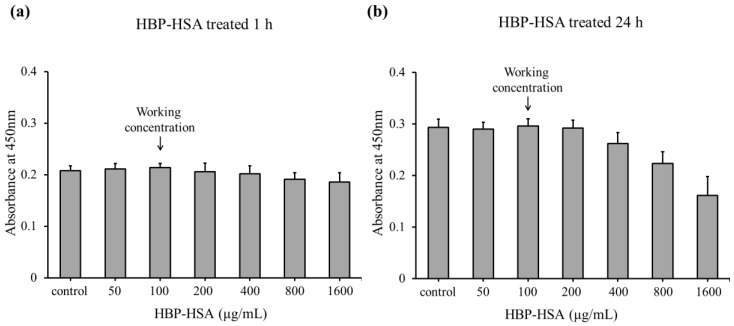
Effect of HBP-HSA on cell viability. HUVECs were seeded in a 96-well plate at a density of 5 × 10^3^ cells/well. Cells were pre-treated with various concentrations of HSA-HBP for 1 h (**a**) and 24 h (**b**), respectively. After culturing at 37 °C for an additional 12 h, the cytotoxicity of HBP-HSA was accessed by CCK-8 assay, and the absorbance of each sample was measured at 450 nm. Arrows indicate the working concentration of HBP-HSA for layer-by-layer heparinization. All experiments were performed three times, and the results are presented as the mean ± standard deviation.

Finally, the effect of the HBP-HSA on cell viability was assessed using the Cell Counting Kit-8 (CCK-8). HUVECs were co-cultured with various concentrations of HBP-HSA conjugate, including an immobilization working concentration of 100 μg/mL for 1 h and 24 h, respectively. As shown in Figure 5a, the results demonstrated that the treatment of HBP-HSA for 1 h had no effect on the proliferation of HUVECs, even at a high concentration of 1600 μg/mL, which was much higher than the working concentration for UFH immobilization. For the 24-h treatment, HBP-HSA started to suppress HUVEC growth at a concentration of 400 μg/mL, which was also much higher than the working concentration for heparinization. Thus, the results indicated that HBP-HSA exhibited excellent cytocompatibility.

## 4. Discussion

Immobilization of heparin or HSA on blood-contacting surfaces provides different sets of benefits. Surface heparinization forms a regional anticoagulant/antiinflammatory environment with a reduced bleeding risk compared with the systemic administration of heparin. The most abundant plasma protein, HSA, is an attractive drug carrier with many advantages, e.g., a long half-life, non-toxicity, non-immunogenicity and high solubility [16]; therefore, it is obvious that the biocompatibility of HSA is superior to avidin or other xenogeneic heparin-binding proteins. Moreover, the albumin coating forms a natural interface between the cell surface and the bloodstream with reduced non-specific interactions, as well as decreased platelet activation/adhesion risks. Therefore, the combination of heparin and HSA for hemocompatible coatings is promising. Several previous studies prepared an electrostatic multilayer of albumin-heparin by utilizing their opposite net charges. However, an acidic environment of pH 4 is required for albumin to interact with negatively charged heparin, and thereby these methods could be detrimental to the survival of cell grafts. By contrast, HBP-HSA binds heparin under physiological conditions, thus avoiding the damages to cells caused by harsh conditions.

By contrast, the surfaces of many medical devices could be heparinized with high efficiency by physical absorption or chemical reactions. Living cells are too fragile for most of the immobilization methods, and thus the efficiency to prepare the first heparin layer on the cell surface is a major constraint to the following layer-by-layer assembly. Cabric et al. covalently modified islet surface with *N*-hydroxysuccinimide (NHS)-biotin, and avidin was then immobilized by reaction with biotin. Finally, heparin was coated by interactions with avidin. This approach is efficient; nevertheless, the random modifications of surface proteins may affect the biological functions of cells. In this study, we synthesized an HBP-PEG-lipid conjugate, and the HBP was anchored onto the cell membrane via the lipid tail of this conjugate, which has already proved harmless to cells [17]. 

The retention of immobilized heparin on the cell surface is also important for the protection of cells. Previous clinical data indicated that IBMIRs were triggered immediately after the cell transplantation and lasted for a few hours to dozens of hours, depending on different cell types [18,19]. Our results revealed that most of the immobilized heparin was retained on the cell surface for 24 h after the heparinization, suggesting that the valid time of heparin coating was sufficient to cover the period of IBMIR.

## 5. Conclusions

Taken together, our preliminary data have demonstrated a simple method to introduce a heparin/HSA multilayer onto the cell surface. This approach has several advantages: firstly, HBP-HSA is cost-effective and easy to prepare; secondly, co-immobilized HSA could potentially provide benefits of albumin coating; finally, HBP-HSA has a remarkable low-cytotoxicity to HUVECs, which could minimize the cell loss caused by in vitro preventive anticoagulant treatment prior to transplantation. Therefore, we anticipate this conjugate could become a promising substitute of heterogeneous heparin-binding proteins for layer-by-layer applications.
